# Stress-Buffer-Hypothesis: blood endocannabinoids in healthy males under standardized psychosocial stress induction and resting condition

**DOI:** 10.1038/s41398-025-03742-4

**Published:** 2025-11-24

**Authors:** Katja Petrowski, Laura Bindila, Benedict Herhaus, Wei Gao, Rupert Conrad

**Affiliations:** 1 Medical Psychology & Medical Sociology, University Medical Center of the Johannes Gutenberg University Mainz, Mainz, Germany; 2 Department of Physiological Chemistry-Lipidomics, Mainz University, Mainz, Germany; 3 School of Psychology, Nanjing Normal University, Nanjing, China; 4 Faculty of Psychology, Chair of Biological Psychology, Technische Universität Dresden, Dresden, Germany; 5 Department of Psychosomatic Medicine and Psychotherapy, University Hospital Muenster, Muenster, Germany

**Keywords:** Diagnostic markers, Human behaviour

## Abstract

The present study investigates the concentrations of the endocannabinoids under standardized psychosocial stress induction (TSST) and a resting condition in healthy males. Hereby, all endocannabinoids were analyzed under a standardized laboratory procedure (arachidonic acid (AA), arachidonoylethanolamide (AEA), isomeres 2-AG arachidonoylglycerol (2-AG) and palitoylethanolamide (PEA)). A total of n = 32 healthy controls (HC) were included in the study. The participants were exposed to the Trier Social Stress Test (TSST) for reliable laboratory stress induction and under rest. Blood samples were taken during the TSST by an intravenous catheter to examine the endocannabinoid (eCB) stress response. There were no significant differences in baseline levels of the parameters between the TSST and the resting condition (p´s > 0.28). ANOVA results indicated a significant effect of time over the six measurements points in all parameters. In the parameter 2-AG and AA a strongly, and in AEA a slightly, significant effect of condition*time could be unveiled. In conclusion, the present study showed that acute psychosocial stress increases plasma endocannabinoids. Further research is required to evaluate the endocannabinoid system in different anxiety disorders to elucidate which patients might benefit from eCB-based therapy.

## Introduction

Stress is recognized as one of the most significant contributing factors to a wide range of mental and physical conditions, including anxiety disorders, depressive episodes, and cardiovascular disease [1–5]. Various types of psychological stressors are distinguished, such as time pressure, isolation, professional or personal life overload, traumatic experiences with intrusions or repeated panic attacks as well as physical stressors. The differentiation between acute and chronic stressors as well as the kind of stressor play a pivotal role in understanding their impact [6]. In order to address the consequences of stress, a more detailed understanding of the regulatory system is necessary.

When humans encounter a stressor, the hypothalamic-pituitary-adrenal (HPA) axis is triggered the human hypothalamus releases corticotropin-releasing hormone (CRH), which in turn stimulates the pituitary gland to release adrenocorticotropic hormone (ACTH), and stimulates the cortisol production in the adrenal glands. Cortisol plays a role in facilitating the release of glucose for energy to cope with the stress response. Regarding the neurobiology of stress, specific brain regions are primarily involved in processing stress and regulating emotions, such as the ventromedial prefrontal cortex (vmPFC), the amygdala, and the hippocampus [7–9]. Interestingly, these brain regions are also known to have a high density of endocannabinoid (eCB) receptors [10, 11].

Research in animal models suggests that the endocannabinoid system (eCS), with its two main eCBs, anandamide (AEA) and 2-arachidonoylglycerol (2-AG), becomes involved in terminating the initial phase of the stress response regulated by the HPA axis [12]. The eCBs are produced “on demand” in the central nervous system due to an increased neuronal activation by stress. The stress-induced rise in CRH in the basolateral amygdala (BLA) in rodent models triggers the activation of fatty acid amide hydrolase (FAAH), the enzyme responsible for breaking down AEA. This breakdown of AEA contributes to the continuation of the stress response. In a subsequent cascade, 2-AG is mobilized to terminate the stress response [13, 14].

Until now, only six studies in humans have investigated the effect of stress on the endocannabinoid system [15–20]. Except for [20] using a thermal heat stressor, all the other studies used the Trier social stressor (TSST [21], or adapted versions such as the Mastrich acute stress test (MAST [22], as a standardized psychosocial stress induction in order to activate the system. Hereby, the focus was on the understanding of the association between stress and the endocannabinoid system in healthy individuals and even first results in depressed and anxiety patients [17, 19]. Even though the stress induction was standardized, the results were not fully consisted. On the one hand, an increase in 2-AG [20], Palmitoylethanolamides (PEAs) and N-acylethanolamines (NAEs) in blood could be observed [16]. On the other hand, no significant increase of 2-AG and AEA could be observed, except in the saliva or only in male participants [18].

The heterogeneity of the literature might be explicable due to the differences in parameters measured for the endocannabinoid system, the handling of the blood and the effect of gender [16, 18]. Even though the studies included a non-stress control task, such as speaking about a neutral topic, or a placebo TSST or MAST as a second condition, the natural fluctuation of the endocanninoids cannot be diminished by the stress effects and as an additional influencing factor.

Endocannabinoids are reported to be stress - sensitive; thus, a correlation with cortisol can be postulated. However, in one study the effect of the stressor could not be observed [19] and, therefore, the full interplay between the endocannabinoid system with the parasympathetic and the sympathetic system needs to be investigated further.

Therefore, the aim of the present study was to investigate the endocannabinoids (arachidonic acid (AA), arachidonoylethanolamide (AEA), isomeres 2-AG arachidonoylglycerol (2-AG), and palitoylethanolamide (PEA)) under a standardized psychosocial stressor only in healthy male participants at a within resting condition. In addition, the blood was to be handled as recommended [23].

## Methods

### Study participants

N = 32 healthy participants were recruited via newspaper advertisements. Inclusion criteria embraced an age between 18 and 65, male sex, and fluency in the German language. Furthermore, the healthy participants were tested for psychological disorders by a standardized interview using the Structured Clinical Interview both for axis I and II [24]. Exclusion criteria involved any kind of psychological diagnoses, medication or substance intake, stressful life events in the previous six months, and severe chronic physical diseases, such as diabetes or cancer. The mean age of the 32 male participants was 24.19 (SD = 4.03) with a mean BMI of 22.83 kg/m² (SD = 1.68). Three of the 29 lastly included participants were smokers (under 10 cigarettes per day). A description of the 32 male participants is given in Table 1. All study participants provided written informed consent, and the study protocol was approved by the local ethics committee of the State Medical Association of Rhineland-Palatinate, Germany (No. 2019-14188).

**Table 1:** Characteristics of the male participants.

	Individuals (N = 32)
*Demographic data*	
Age (years), M (SD)	24.19 (4.03)
Body mass index, M (SD)	22.83 (1.68)
Smoking, n (%)	6 (19)
FFKA - Total activity (min/week), M (SD)	1554 (1051)
FFKA – Sport activity (min/week), M (SD)	443 (566)
*Psychological Assessment*	
SCL Global Severity Index, M (SD)	0.34 (0.29)
PSS, M (SD)	21.94 (6.53)
BDI, M (SD)	5.53 (4.67)
TICS-SCSS, M (SD)	11.59 (6.16)
ASI, M (SD)	5.13 (4.72)
STAI-T, M (SD)	43.13 (5.00)

*ASI* anxiety sensitivity index; *BDI* Beck depression inventory; *FFKA* Freiburg questionnaire on physical activity, *PSS* perceived stress scale, *SCL* Symptom-Check-List-90-R, *SCSS* subscale of chronic stress, *STAI* spielberger state-trait anxiety inventory, *TICS* trier inventory of chronic stress, *T* trait.

### Procedures

Data collection of the 32 male healthy participants was conducted from May 2019 to July 2020. The participants underwent a stress and rest conditions on different days over a total period of seven days. The test order of the two conditions (stress and rest) was randomized, and the start of each condition was scheduled between 2:00 p.m. and 5:00 p.m. to minimize the impact of significant circadian cortisol level fluctuations. The participants were instructed not to eat, drink, or smoke for up to two hours before the testing session to ensure uniform testing conditions and prerequisites for all. Forty-five minutes before the first blood collection, the intravenous cannula was inserted to avoid a pain-induced endocannabinoids and cortisol release. The standardization of the stress induction was based on the Trier Social Stress Test (TSST) introduced by [21]. The TSST is designed to induce acute psychosocial stress in a controlled laboratory environment. This stress protocol involves a social-evaluative scenario that includes a five-minute simulated job interview followed by a five-minute mental arithmetic task, both of which are performed in front of two evaluators. A detailed description and assessment of the effectiveness of this psychosocial stress protocol can be found in [25]. During the rest condition, the participants were given the opportunity to read magazines. The experimental protocol began with a 15-min pre-session. The first blood sample was collected one minute before the start of the 15-min conditions (stress and rest). Three minutes after the onset of the corresponding condition, cognitive assessment was assessed with the Primary Appraisal Secondary Appraisal (PASA; 28). Immediately after both conditions, a second blood sample was collected and self-reported stress perception was measured with the visual analog scale (VAS). During the recovery phase, the participants were reposing in supine position on a surgery bed, while seven further blood samples (+10, +20, +30, +45, +60, +75 and +105 min) were collected.

### Blood analytics

To assess plasma endocannabinoids concentrations, blood samples were collected in monovettes containing containing ethylenediaminetetraacetic acid (EDTA) (Sarstedt, Nümbrecht, Germany). Following collection, the EDTA monovettes were promptly centrifuged at 4 °C and 2000 g for 10 min. The endocannabinoid concentrations of arachidonic acid (AA), arachidonoylethanolamide (AEA), isomeres 2-AG arachidonoylglycerol (2-AG) and palitoylethanolamide (PEA) were determined. Details regarding the extraction and analysis of endocannabinoids can be found in previously described protocols [23]. For the determination of serum cortisol levels, blood samples were obtained in serum gel monovettes (S-Monovette® 9 ml Z, Sarstedt, Nümbrecht, Germany). These monovettes were left at room temperature for 30 min to facilitate blood coagulation. Upon coagulation, the serum monovettes were centrifuged for 10 min at 2500 g and 20 °C. Serum cortisol concentrations were assessed using a commercially available enzyme-linked immunosorbent assay (ELISA) kit.

### Psychological and clinical measures

The psychological and general status of the participants was measured by means of six instruments: (1.) The Symptom-Check-List-90-R (SCL) [26] which is a self-report instrument for assessing psychological and physical impairments, which consists of 90 items with a five-point rating scale (Cronbach’s Alpha-coefficient in the current sample α = 0.95). (2.) The Perceived Stress Scale (PSS) constructed by [27] that measures the perception of stress and consists of 14 items with a five-point Likert scale ranging from 1 (never) to 5 (very often). The reliability (Cronbach’s Alpha-coefficient) of the 14 items in the current sample was α = 0.75. The (3rd) Beck Depression Inventory (BDI) [28] is used to assess severity of depressive symptoms based on 21 items (total score range: 0 - 63). The reliability (Cronbach’s Alpha-coefficient) of the 21 items in the current sample was α = 0.76. (4.) The Trier Inventory for Chronic Stress (TICS) [29] that measures the participants’ perceived chronic stress during the previous three months (Cronbach’s Alpha-coefficient in the current sample α = 0.92). (5.) The Freiburg Physical Activity Questionnaire (FFKA) [30] was used to assess the different types of daily activity. The reliability (Cronbach’s Alpha-coefficient) of the 21 items in the current sample was α = 0.75. (6.) The tendency to fear anxiety - related sensations and personal sensitivity to fearing symptoms (somatic and cognitive) is assessed by the Anxiety Sensitivity Index (ASI) [31] with 16 items (Cronbach’s Alpha-coefficient in the current sample α = 0.79). (7) The Spielberger State-Trait Anxiety Inventory (STAI) [32] was used to assess the trait anxiety (Cronbach’s Alpha-coefficient in the current sample α = 0.91).

The Primary Appraisal Secondary Appraisal (PASA) [28] was measured three minutes after the start of each condition to assess the cognitive appraisal processes. The questionnaire consists of 16 items rated on a six-point Likert scale (1 = strongly disagree to 6 = strongly agree), allowing for the calculation of Primary Appraisal, Secondary Appraisal, and the Stress Index. Before and after both conditions, the temporary emotional state of anxiety was assessed by the state anxiety scale (20 items) of the Spielberger State-Trait Anxiety Inventory (STAI) [32]. The visual analogous scale (VAS) was used to assess self-reported feelings of stress after both conditions. This ranges from 0, which corresponds to no stress at all, to 100, which equates to maximum stress.

### Statistical analyses

The software program used to analyze the data was SPSS Statistics version 27 (IBM, Chicago, IL, USA). A power analysis with the G*power program (version: 3.1.9.2.) [33] showed that for a medium effect size of Cohen´s f =0.25, two testing conditions (TSST and resting condition), in each condition at least n = 6 repetitions, significance level of p = 0.05 and power of 95% (1-ß = 0.95), a total sample size of n = 28 participants for ANOVA-repeated measures (within-between interaction) are needed. The data were analyzed according to the normality of distributions and were, in case of not normally distributed data, subjected to logarithm naturalis transformations.

Firstly, the effects of the TSST and the resting condition over six measurement points for the endocannabinoids as well as over nine measurement points for the cortisol concentration were analyzed by the ANOVA for repeated measurements to reveal possible main effect of condition or time and a possible time x condition interaction. The assumption of sphericity was controlled by Mauchly’s test. Whenever necessary, the ANOVA results were corrected by Greenhouse-Geisser. In addition, the delta between peak (either +1, +5, +10, +20, +30, +45, +60, +75, +105 min sample) and baseline (Δ Peak-Base) was calculated [34]. Differences of the calculated values between both conditions were tested by the t-test for dependent means.

Secondly, the success of the stress induction on the cognitive appraisal processes (PASA) and the acute self-reported stress perception (VAS) were tested by the dependent t-test. To reflect the effect of the stress condition on the temporary emotional state of anxiety (STAI-S), ANOVA for repeated measurements with within-factor condition (stress vs. rest) and time (pre vs. post) was analyzed.

Thirdly, Pearson’s correlations were calculated to quantify the relationship between baseline the values of endocannabinoids, cortisol, and psychological (BDI, ASI, PSS, and TICS-SCSS) measures.

Fourthly, the association between acute stress-induced peak values of endocannabinoids/cortisol and subjective acute stress appraisal was tested using Pearson’s correlation test.

## Results

### Endocannabinoid response to acute stress compared to rest

**Fig. 1:**
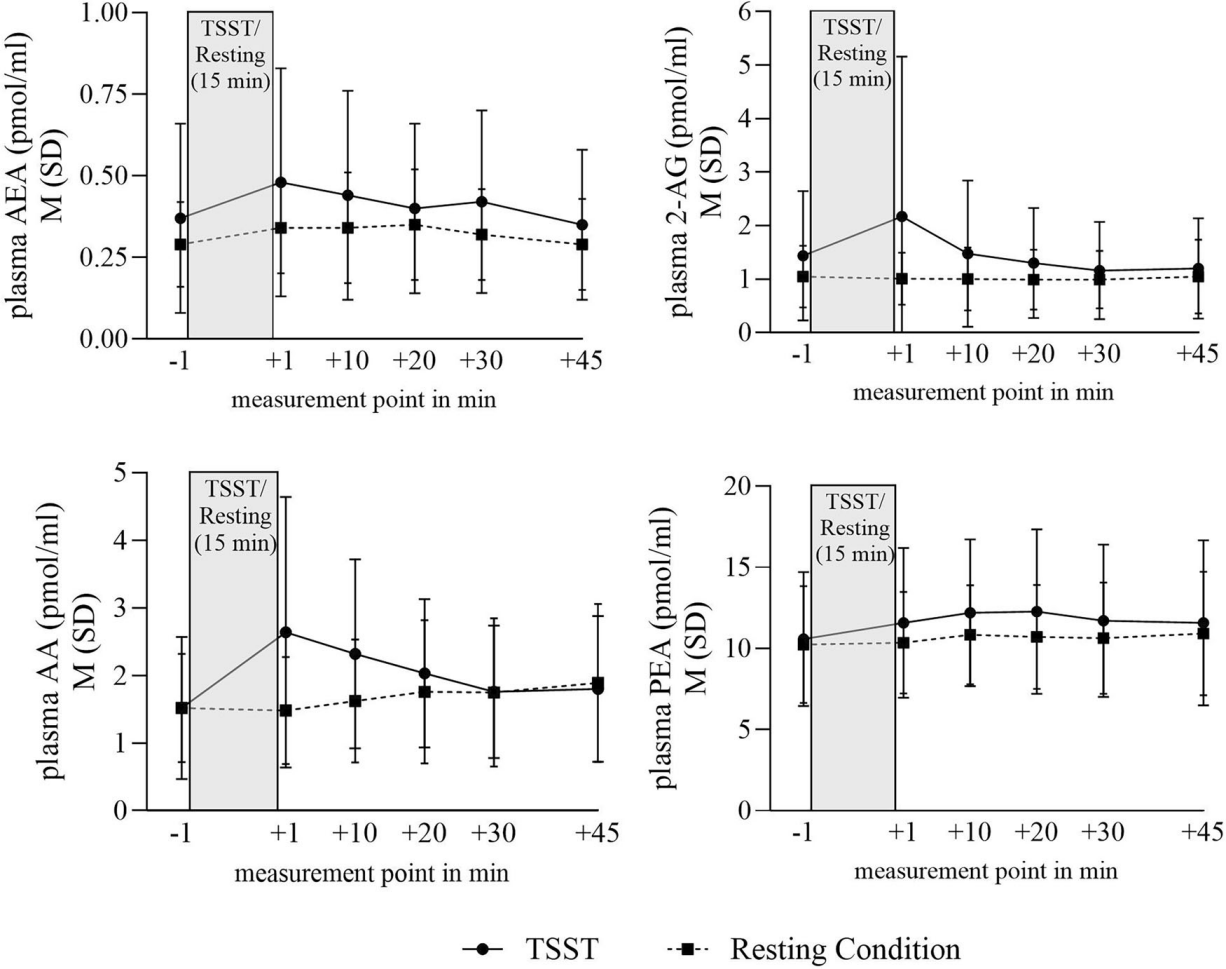
Endocannabinoid concentration AEA, 2-AG, PEA, AA during Trier Social Stress Test and resting condition in health males.

Figure 1 presents the AEA concentration, 2-AG concentration, PEA concentration, and AA concentration over the six measurement points during the resting condition and the psychological stress induction (TSST). There were no significant differences in baseline levels of the parameters between the TSST and the resting condition (p´s > 0.28). ANOVA results (Table 2) indicated a significant effect of time over the six measurements points in all parameters. In the parameter 2-AG, a significant main effect of condition could be unveiled. Furthermore, ANOVA´s demonstrated an interaction effect time x condition in AEA, 2-AG, and AA (see Table 2).

**Table 2:** ANOVA results for endocannabinoids AEA, 2-AG, PEA, AA.

Outcome	Condition	ANOVA time	ANOVA condition	ANOVA condition*time
AEA^a^	TSST Resting	*F* = 10.310, *df* = 5, ***p*** ≤ **0.001,** *η*^*2*^ = 0.250	*F* = 3.847, *df* = 1, *p* = 0.06	*F* = 2.752, *df* = 3.755, ***p*** ≤ **0.05,** *η*^*2*^ = 0.082
2-AG^a^	TSST Resting	*F* = 11.776, *df* = 3.170, ***p*** ≤ **0.001,** *η*^*2*^ = 0.275	*F* = 4.425, *df* = 1, ***p*** ≤ **0.05,** *η*^*2*^ = 0.125	*F* = 8.825, *df* = 2.938, ***p*** ≤ **0.001,** *η*^*2*^ = 0.222
PEA^a^	TSST Resting	*F* = 3.685, *df* = 3.771, ***p*** ≤ **0.01,** *η*^*2*^ = 0.106	*F* = 1.647, *df* = 1, *p* = 0.21	*F* = 1.032, *df* = 3.887, *p* = 0.39
AA^a^	TSST Resting	*F* = 16.267, *df* = 1.003, ***p*** ≤ **0.001,** *η*^*2*^ = 0.344	*F* = 3.021, *df* = 1, *p* = 0.09	*F* = 20.240, *df* = 2.429, ***p*** ≤ **0.001,** *η*^*2*^ = 0.395

ANOVA results are presented as *F*-values, degrees of freedom (*df*), *p*-values, and effect size Eta squared *η*^*2*^ (if significant).

*AA* arachidonic acid, *AEA* arachidonoylethanolamide, *PEA* palitoylethanolamide, *TSST* trier social stress test, *2-AG* 2-arachidonoylglycerol.

^a^values were subjected to natural log transformations due to not normally distributed data.

With regard to the derived parameter Peak, significant differences between the TSST and the resting condition could be unveiled in AEA, 2-AG, and AA with higher values for the stress condition (see Table 3). Furthermore, there was a significant difference in the derived parameter Delta of 2-AG and AA with higher values for the TSST condition.

**Table 3:** Derived parameter for endocannabinoids AEA, 2-AG, PEA, AA.

		TSST		Resting			
Derived parameter Peak		M	SD	M	SD	*t* (1,31)	*p*
Endocanna-binoids (pmol/ml)	AEA^a^	0.54	0.34	0.41	0.17	2.236	**≤0.05** (*d* = 0.40)
	2-AG^a^	2.28	2.96	1.26	0.71	−3.488	**≤0.05** (*d* = 0.43)
	AA^a^	2.78	1.95	2.08	1.16	−5.740	**≤0.05** (*d* = 0.40)
	PEA^a^	14.13	5.19	12.66	3.42	−3.589	0.15

Derived parameter Delta		M	SD	M	SD		
Endocanna-binoids (pmol/ml)	AEA^a^	0.18	0.14	0.12	0.10	1.830	0.08
	2-AG^a^	0.84	1.86	0.21	0.28	−1.635	**≤0.05** (*d* = 0.39)
	AA^a^	1.26	1.00	0.56	0.67	2.080	**≤0.001** (*d* = 0.78)
	PEA^a^	3.56	2.74	2.41	2.32	−0.525	0.15

T-test results are presented as t-values, degrees of freedom (*df*), *p*-values, and effect size Cohen's d (if significant).

*AA* arachidonic acid, *AEA* arachidonoylethanolamide, *M* mean, *PEA* palitoylethanolamide, *SD* standard deviation, *TSST* trier social stress test, *2-AG* 2-arachidonoylglycerol.

^a^values were subjected to natural log transformations due to not normally distributed data.

### Psychological responses to stress induction versus rest

Table 4 presents state anxiety, cognitive appraisal and perceived stress to the stress induction versus the resting condition. There was a significant time x condition interaction effect (*F*_(1, 30)_ = 20.936, *p* ≤ 0.001, *η*^*2*^ = 0.411) in the temporary emotional state of anxiety with higher values after the stress induction compared to the resting condition. Furthermore, all primary, secondary and tertiary scales of the anticipatory cognitive appraisal of stress (PASA) showed a significant difference between stress and resting condition (see Table 4). In addition, all participants exhibited a higher stress perception in the VAS after the stress induction compared to rest (*t* (31) = 7.811, *p* ≤ 0.001, *d* = 1.38).

**Table 4:** Influence of stress condition on state anxiety, cognitive appraisal and perceived stress.

		TSST		Resting Condition			
		M	SD	M	SD	*t* (1,31)	*p*
STAI	State_PRE_	34.74	7.52	33.39	5.17	1.319	0.20
	State_POST_	40.94	9.11	31.26	6.04	6.092	**≤0.001** (*d* = 1.09)
PASA	Threat	3.06	1.06	1.48	0.80	8.351	**≤0.001** (*d* = 1.48)
	Challenge	4.07	0.79	2.26	0.95	8.875	**≤0.001** (*d* = 1.57)
	Self concept	3.88	1.03	4.53	0.88	−2.766	**≤0.01** (*d* = −0.49)
	Control expectancy	4.21	0.97	4.60	0.70	−2.336	**≤0.05** (*d* = −0.41)
	Primary appraisal	3.56	0.81	1.87	0.77	10.361	**≤0.001** (*d* = 1.83)
	Secondary appraisal	4.04	0.87	4.57	0.63	−3.228	**≤0.01** (*d* = −0.57)
	Stress index	−0.48	1.39	−2.70	1.24	8.049	**≤0.001** (*d* = 1.42)
VAS		57.77	9.53	42.07	11.05	7.811	**≤0.001** (*d* = 1.38)

T-test results are presented as t-values, degrees of freedom (*df*), *p*-values, and effect size Cohen’s d (if significant).

*PASA* primary appraisal secondary appraisal, *S* state, *STAI* spielberger state-trait anxiety inventory, *VAS* visual analogue scale.

### Baseline intercorrelations between endocannabinoids, cortisol, and psychological measures

No significant correlation could be found between baseline values of endocannabinoids, cortisol, and psychological (BDI, ASI, PSS, and TICS-SCSS) measures (see Table 5). The psychological measurements showed correlations as expected.

**Table 5:** Pearson’s correlations (r) between baseline endocannabinoids, baseline cortisol, and psychological measures.

		ln baseline endocannabinoids				baseline cortisol	Psychological measures				
		AEA	2-AG	AA	PEA		STAI-T	ASI	BDI	PSS	TICS-SCSS
ln baseline endocannabinoids	AEA	1.00									
	2-AG	0.00, *p* = 0.99	1.00								
	AA	0.46, *p* ≤ 0.01	−0.03, *p* = 0.86	1.00							
	PEA	0.26, *p* = 0.15	0.12, *p* = 0.53	0.22, *p* = 0.22	1.00						
baseline cortisol		−0.03, *p* = 0.86	−0.23, *p* = 0.21	0.02, *p* = 0.92	−0.26, *p* = 0.16	1.00					
Psycho-logical measures	STAI-T	0.11, *p* = 0.56	0.14, *p* = 0.44	0.24, *p* = 0.19	0.07, *p* = 0.69	−0.33, *p* = 0.07	1.00				
	ASI	0.02, *p* = 0.91	−0.12, *p* = 0.51	0.09, *p* = 0.63	0.05, *p* = 0.78	−0.24, *p* = 0.20	0.38, *p* ≤ 0.05	1.00			
	BDI	−0.12, *p* = 0.50	−0.21, *p* = 0.25	0.04, *p* = 0.82	0.02, *p* = 0.91	0.09, *p* = 0.62	0.27, *p* = 0.14	0.44, *p* ≤ 0.05	1.00		
	PSS	−0.12, *p* = 0.51	−0.04, *p* = 0.82	0.05, *p* = 0.78	−0.14, *p* = 0.44	−0.09, *p* = 0.62	0.32, *p* = 0.07	0.60, *p* ≤ 0.001	0.49, *p* ≤ 0.01	1.00	
	TICS-SCSS	−0.06, *p* = 0.74	0.04, *p* = 0.83	0.01, *p* = 0.95	−0.03, *p* = 0.89	−0.24, *p* = 0.19	0.46, *p* ≤ 0.01	0.47, *p* ≤ 0.01	0.33, *p* = 0.07	0.65, *p* ≤ 0.001	1.00

Data are presented as coefficient, *p* values.

*AA* arachidonic acid, *AEA* arachidonoylethanolamide*, ASI* anxiety sensitivty index; *BDI* Beck depression inventory, *PSS* perceived stress scale, *SCSS* subscale of chronic stress, *STAI* spielberger state-trait anxiety inventory, *TICS* trier inventory of chronic stress, *T* trait.

### Acute stress-induced peak values of endocannabinoids/cortisol and subjective acute stress appraisal

As shown in Table 6, there was a significant correlation between the stress-induced peak level of AA and the tertiary scale stress-index of the anticipatory cognitive appraisal of stress (r(30) = 0.45, *p* ≤ 0.01). In addition, a marginally significant correlation could be observed between the stress-induced peak level of AA and a difference between the temporary emotional state of anxiety before and after the stress induction (r(30) = 0.34, *p* = 0.06).

**Table 6:** Pearson’s correlations (r) between acute stress-induced peak values of endocannabinoids/cortisol and subjective acute stress appraisal.

		stress-induced peak ln endocannabinoids				stress-induced peak cortisol	Subjective acute stress appraisal		
		AEA	2-AG	AA	PEA		PASA stress-index	VAS	STAI-S DeltaPost-Pre
stress-induced peak ln endocannabinoids	AEA	1.00							
	2-AG	0.70, *p* ≤ 0.001	1.00						
	AA	0.74, *p* ≤ 0.001	0.37, *p* ≤ 0.06	1.00					
	PEA	0.54, *p* ≤ 0.001	0.67, *p* ≤ 0.001	0.51, *p* ≤ 0.01	1.00				
stress-induced peak cortisol		0.04, *p* = 0.84	−0.04, *p* = 0.83	0.01, *p* = 0.95	−0.04, *p* = 0.85	1.00			
Subjective acute stress measures	PASA stress-index	0.29, *p* = 0.11	0.02, *p* = 0.94	0.45, *p* ≤ 0.01	0.04, *p* = 0.82	0.12, *p* = 0.52	1.00		
	VAS	−0.09, *p* = 0.62	−0.23, *p* = 0.21	>0.01, *p* = 0.99	0.00, *p* = 1.00	−0.05, *p* = 0.78	0.14, *p* = 0.44	1.00	
	STAI-S DeltaPost-Pre	0.28, *p* = 0.13	0.03, *p* = 0.88	0.34, *p* = 0.06	0.28, *p* = 0.12	0.19, *p* = 0.31	0.19, *p* = 0.31	0.30, *p* = 0.09	1.00

Data are presented as coefficient, *p* values.

*AA* arachidonic acid, *AEA* arachidonoylethanolamide*, PASA* primary appraisal secondary appraisal, *S* state, *STAI* Spielberger state-trait anxiety inventory, *VAS* visual analogue scale.

## Discussion

To the best of our knowledge, this is the first study analyzing stress-induced blood concentrations of endocannabinoids and related N-acylethanolamines (2-AG; AEA; AA; PEA) compared to a resting condition with a highly standardized centrifugation protocol in a male sample [23].

In the present study, there were no significant differences in the baseline levels of the parameters between the TSST and the resting condition (p´s > 0.28). ANOVA results indicated a significant effect of time over the six measurement points in all parameters. In parameter 2-AG a strongly and in AEA and AA a slightly significant main effect of condition could be unveiled with a significant interaction effect time x condition in 2-AG, AEA, and AA.

The significant increase in 2-AG is in line with the significant increase in 2-AG in male participants by [18] and by [20], even though they used a heat stimulus for stress induction. The increase in AEA through stress induction in male participants is also in line with [18]. However, the results on PEA with a stress-induced increase could not be observed in the present study as by [16]. In addition, the stress - induced changes of AA compared to the resting condition in the present study had not yet been observed before, to our knowledge.

Besides the stress induced course of the endocannabinoids, the interplay with other stress - related markers are of interest. Concerning subjective stress evaluation, there was a significant correlation between the stress-induced peak level of AA and the tertiary stress-index of the anticipatory cognitive appraisal of stress and a marginally significant correlation with the course of the emotional state of anxiety from before to after. Concerning cortisol concentrations, however, no associations were found between the endocannabinoids and cortisol even though stress increases the concentrations of the endocannabinoids– and cortisol, which is in line with earlier results reported by [16]. This absence of a correlation aligns with the stress-buffering function of the endogenous cannabinoid system, which primarily modulates sympathetic nervous system activity, as evidenced by norepinephrine release, rather than influencing the HPA-axis activation represented by cortisol [35, 36].

To gain a deeper understanding, it is crucial to comprehend their metabolic processes and pathways. The production of 2-AG primarily stems from inositol phospholipids, proceeding through diacylglycerol via phospholipase C and diacylglycerol lipase. The presynaptic enzyme monoacylglycerol lipase (MAGL) plays a pivotal role in metabolizing 2-AG. In contrast, AEA is synthesized alongside significant quantities of other N-acyletanolamines like PEA, originating from N-acyl-phosphatidylethanolamines (NAPE) through calcium-dependent N-acyltransferase and NAPE-hydrolyzing phospholipase [37, 38].

While AEA and 2-AG serve as the primary endogenous agonists for CB1 and CB2 receptors, other N-acylethanolamines do not directly bind to cannabinoid receptors. CB1 receptor agonists can elicit both anxiolytic and anxiogenic effects, influenced by factors such as dosage, route of administration, variations in CB1 receptor sensitivity across different brain regions, and the aversive nature of the behavioral testing paradigm employed [39]. The systemic or central elevation of anandamide levels (AEA), achieved through the pharmacological inhibition of FAAH is well-documented to produce anxiolysis, particularly in more aversive conditions [40]. The above outlined mechanisms can be supported by positive associations between anandamide - produced N-acylethanolamines, 2-AG and self-reported anxiety symptoms in patients with panic disorder [19].

The inconsistency in the above-mentioned studies in healthy controls is striking. The variations observed could be attributed, in part, to specific characteristics of the study samples. Notably, there were significant differences in the sex distribution across the samples [16]: had N = 71, with 71% female [17]; included N = 15, with 100% female participants [41]; featured N = 10, with 70% female; in our study [19], N = 26, with 46% female participants. Despite these disparities in sex distribution, they offer only a partial explanation for the inconsistencies, especially if there is a sex-specific sensitivity to the endocannabinoid-elevating effects of TSST, as proposed by [16]. Of potentially greater significance might be endocrinological variations associated with the menstrual cycle [42]. In two studies [17, 41] the cycle phase was not controlled for and [16] tested female participants in the luteal as well as the follicular phase. Therefore, the present study tested the main mechanism based on an exclusive male sample, which leads to a limiting generalizability.

The strength of the study is the high standardization through a healthy male sample without any psychological disorders (lifetime, based on SCID), a highly standardized psychosocial stressor, and a highly standardized blood processing procedure. However, several limitations of our study need to be considered. First, endocannabinoids were collected from blood, and the correlation between blood and brain endocannabinoid levels is difficult to establish. Second, the results based on an exclusively male sample in order to exclude the influence of the menstrual cycle. Therefore, the results might not be transferable to healthy female participants with their different phases of the menstrual cycle. Third, the young age of the sample is limiting for the generalizability since the mean age of the German population is higher. Thus, the generalizability is limited.

The task for future research at the current state of knowledge should be to broaden the understanding of the role of the endocannabinoid system in healthy individuals as well as of different anxiety disorders to elucidate which patients might benefit from EC-based therapy in the future. Knowledge of the modulation of the ECS activity has shown itself as promising in the treatment of diseases and pathological conditions, such as, e.g., glaucoma, neuropathic conditions, and osteoporosis.

## Data Availability

The raw data supporting the conclusions of this article will be made available by the corresponding author upon reasonable request.
